# Correction to: The role of connexin proteins and their channels in radiation-induced atherosclerosis

**DOI:** 10.1007/s00018-021-03811-z

**Published:** 2021-03-29

**Authors:** Raghda Ramadan, Sarah Baatout, An Aerts, Luc Leybaert

**Affiliations:** 1 Radiobiology Unit, Belgian Nuclear Research Centre (SCK CEN), Mol, Belgium; 2 Department of Basic and Applied Medical Sciences, Physiology group, Ghent University, Ghent, Belgium; 3 Department of Molecular Biotechnology, Ghent University, Ghent, Belgium

## Correction to: Cellular and Molecular Life Sciences 10.1007/s00018-020-03716-3

The article The role of connexin proteins and their channels in radiation-induced atherosclerosis, written by Raghda Ramadan, Sarah Baatout, An Aerts and Luc Leybaert was originally published electronically on the publisher’s internet portal (currently SpringerLink) on 3rd January 2021 without open access. With the author(s)’ decision to opt for open choice, the copyright of the article changed on 16th March 2021 to © The Author(s) 2021 and the article is forthwith distributed under the terms of the Creative Commons Attribution 4.0 International License (http://creativecommons.org/licenses/by/4.0/), which permits use, duplication, adaptation, distribution and reproduction in any medium or format, as long as you give appropriate credit to the original author(s) and the source, provide a link to the Creative Commons license and indicate whether changes were made.

The original article has been corrected.

